# The Principles of Surgery

**Published:** 1845-04

**Authors:** 


### ART. XII.

*The Principles of Surgery.* By JAMES MILLER, F.R.S.E., Professor of Surgery in the University of Edinburgh, Surgeon to the Royal Infirmary, &c. &c.—*Edinburgh,* 1844. 8vo, pp. 716.

IT would be extremely difficult, indeed almost impossible, to give a satisfactory review of such a book as Mr. Miller's in a Journal conducted on the plan that ours is. Mr. Miller's work purports to be an epitome of the entire body of doctrine on which the whole of the modern practice of surgery is based, and to attempt to analyse it in detail would therefore involve a commentary on every important subject comprised within the term "Principles of Surgery;" but such a commentary would occupy not a portion of one number, but several consecutive numbers of our Journal. We might, it is true, set forth our author's table of contents, and enumerate the several subjects treated of in the subsections of each chapter, but this would be to offer to our readers the dry bones without the flesh, to tell that Mr. Miller had written on such and such subjects, without giving any information as to what he had written about them. It might seem open to us to specify whatever new views are contained in the work; but Mr. Miller does not profess to teach novelties; his object has been to give a condensed account of the most generally admitted doctrines; to use his own words, "a concise Exposition of the Science of Modern Surgery." Every chapter of the work indeed would afford ample subject for an article in this Journal, and as occasion arises we shall not fail to refer to Mr. Miller's views, so far as may be necessary when separately discussing any of the numerous subjects collectively treated of in his volume. In our opinion this is the only way in which a systematic work professing to embrace the entire groundwork of an extensive department of science can be profitably dealt with by the reviewer; and we shall consequently, for the present, content ourselves with expressing our opinion of the manner in which Mr. Miller has executed his arduous task, and extracting a few passages which may, perhaps, though imperfectly, enable our readers to form some estimate of how far the judgment we have formed of the work is well founded, or the reverse.

We have no hesitation in pronouncing a decidedly favorable verdict on Mr. Miller's book. We owe him indeed no small thanks for the mere conception of the plan of devoting his work to the principles of surgery. The tendency of surgery has been too long decidedly mechanical, a striving after minute modifications of methods of operating and peddling contrivances in surgical apparatus, rather than the profound study of the principles of surgery, without a knowledge of which the most dexterously performed operation is liable to fail. Of late years there has, doubtless, been a wholesome reaction in this respect—a reaction commenced, and as yet chiefly furthered by British surgeons; and we deem it a satisfactory evidence of this favorable tendency in the surgery of the day that Mr. Miller, who, we believe, is still young, has preferred writing on the principles of surgical science—those principles which alone can make surgery a science—to concocting a book on the more popular and showy, but after all mechanical departments, which it is even yet rather too much the fashion to attach rather an undue importance to.

As an example of the value of a correct appreciation of the principles of surgery in the treatment of disease, we may refer to the recent important improvement in the treatment of popliteal aneurism, by substituting compression of the femoral artery for ligature of that vessel. We take this example, not as the best by any means that we could select, but because it relates to one of the very few subjects admitting of anything like novelty contained in Mr. Miller's work; and consequently the following extract, being Mr. Miller's section on the cure of aneurism by "*pressure*, without incision," besides a fair example of Mr. Miller's style and manner of handling his subject, will in itself prove interesting to most and instructive to some of our readers.

"In ancient times, the surgeon who was afraid to cut into the aneurism, and take his chance of arresting the flow of blood, had recourse to direct and energetic compression of the part, with the hope of cure. The name of Guattani is chiefly associated with the practice. Local sloughing, suppuration, or ulceration, with severe constitutional disturbance, yet with an unclosed artery and aneurism, resulted more frequently than the cure. Subsequently to the establishment of the Hunterian operation, its principle was extended to the mode of treatment by pressure; this being applied, not to the tumour itself, nor in its immediate vicinity, but at some distance, at a part such as would be selected for Hunterian deligation, in the hope of the arterial tissue there being in a sound condition. This method was made trial of by Dubois, A. Cooper, Blizard, &c. but with no satisfactory issue. The pressure was continued and severe, their object being to keep the tube close and impervious at that point, and by plastic deposit to obtain its complete consolidation. The result was, the occurrence of great pain and constitutional disturbance; followed by inflammation, ulceration, or sloughing of the compressed parts; exposing, or perhaps including, the vessel. The practice found no favour with the general profession. Lately, however, the treatment by pressure has been revived, in a more scientific form, and with a better success; conducted rather as if itself was not the agent of cure, but only the means whereby the spontaneous cure may be originated and favoured. The pressure is made at a Hunterian site, as before, but it is neither constant nor severe. By means of a compressor, such as invented by Crompton and Signorini, or any other suitable apparatus, a moderate degree of pressure is applied to the vessel at a point where its coats may be expected to be sound, and consequently not prone to ulcerate from slight causes. This is maintained so long as it can be conveniently borne by the patient, but no longer. So soon as the uneasy sensations become at all intense, with swelling and numbness of the limb, and throbbing in the part, the pressure is either slackened or altogether removed. After a time, the parts having recovered, it is reapplied; again it is removed; and thus, by its occasional and modified use, the disasters formerly attendant on the treatment by pressure may be altogether avoided. At the same time, the circulation in and near the aneurism is decidedly moderated, so as to favour solidification. The tumour is not only arrested in its growth, but begins to diminish; its pulsation is less, and its dimensions contract; it feels harder and less compressible; ultimately, pulsation wholly disappears, and induration is complete; absorption then advances, and the obliterative cure is obtained, with or without a pervious condition of the vessel. But the pressure is not trusted to alone. The same preparatory treatment is necessary as before the operation by ligature; and throughout the whole period of treatment absolute repose with recumbency is maintained, as well as antiphlogistic regimen, and all other means likely to favour the desired beneficial change. Also, the limb below the compressed point must be uniformly and equally supported by bandaging, lest passive congestion and œdema supervene; and this pressure may from time to time be somewhat increased on that part of the limb which includes the aneurismal tumour. Let no haste be indulged in. The process is necessarily one of weeks, not of days; gradual, not sudden; interrupted, not continuously progressive. The pressure requires to be neither great nor constant, for we do not desire obliteration, even temporary, of the arterial tube there; it is sufficient to moderate, not essential to obstruct, the flow. And only by a constant remembrance that such are the principles of cure by this means, will the pressure be so leisurely and prudently conducted, as to ensure avoidance of the disaster to which compression is liable. The advantage of such a mode of treatment, when properly conducted, is immunity from ulceration and hemorrhage; the disadvantages are, the protracted period, and ultimate uncertainty, of cure. If improperly conducted, it is in every point of view inferior to the ligature; less certain of cure, and even more certain of danger at the selected part of the vessel. Even skilfully managed, it is obviously less capable of general application; there being not a few systems possessed of an intolerance of pressure, even when modified and occasional. The improved revival, however, is as yet but in its infancy. In the hands of Liston, Cusack, Hutton, and others, it has already succeeded. But a wider experience is still required ere surgical opinion can be at rest upon the question. The leading points of the system, it may be again stated, are:—the pressure is at some distance from the tumour, moderate, and only occasional; it is not necessary, and it is not our object, to obliterate the vessel at the compressed point; in other respects the same treatment is followed out, regarding both part and system, as in the favouring of spontaneous cure without any surgical interference." (pp. 457-9.)

The foregoing extract contains an accurate statement and a candid and judicious appreciation of the facts known at the time Mr. Miller wrote, but several cases that have occurred since, strengthen the hope that the treatment of popliteal aneurism by compression will become applicable, if not in every case, at all events in most cases. Dr. Cusack communicated to the Surgical Society of Ireland a case, published in the 'Dublin Medical Press' last February, which though apparently a most unfavorable one for treatment by compression, was nevertheless radically cured in a few days by that method. The aneurismal tumour was very large and had given way, so that it communicated with a secondary sac containing perfectly fluid blood so very thinly covered, being in fact almost subcutaneous, that great apprehensions were entertained that it might burst; the tumour moreover was extremely painful, and the patient was at first so intolerant of pressure that it could not be maintained on any one point for many *consecutive minutes.* Under these unpromising circumstances two instruments were employed, one being tightened so as to moderate the current of blood through the femoral artery without completely arresting it, while the other was relaxed; directly the pressure of the former instrument became inconvenient it was loosened, and the other was brought into play. By thus alternating the action of the two instruments, and by shifting their position (as the pressure, moderate though it was, became intolerable at one point) over the entire space between the pubis and the margin of the tumour, pulsation was completely and permanently arrested in the aneurism by the third day. We thus see that Mr. Miller's estimate of the disadvantages of the treatment of popliteal aneurism by compression, though fully justified by the state of our knowledge at the time he wrote, may probably have to be materially modified. Dr. Cusack's case, just referred to was, it must be admitted, a most unfavorable one for treatment by compression, and yet a cure was effected by that method, not more slowly, but very much more rapidly, than could have been accomplished by ligature of the artery. We are also aware of other cases, indeed have witnessed some of them, in which the treatment by compression was attended with an equally satisfactory, though not certainly so prompt a result; but as those cases have not yet been published, we cannot further allude to them. We may just observe, however, that the rule laid down by Mr. Miller, that "the limb below the compressed point must be uniformly and equally supported by bandaging, lest passive congestion and œdema supervene," &c., is by no means generally true, and in some instances may be advantageously deviated from. In Dr. Cusack's case, above noticed, no bandage was applied to the limb; and in another case, not published, which we witnessed, the limb was left unbandaged, not only without inconvenience, but to the great comfort of the patient; in neither case did the pressure produce œdema, which will not indeed commonly occur, if the compressing instrument is, as it should be, so contrived and applied that it shall bear on two opposed surfaces of the limb without constricting too great a portion of its circumference.

A succinct analysis of Mr. Miller's views respecting inflammation, might seem to afford a good opportunity of testing the value of his work, but we find ourselves precluded from adopting this plan by the fact, that he has done us the honour to adopt, almost entirely, the opinions recently put forward in this Journal, and to which he makes due reference. In the more practical parts of the chapter we have noticed a few errors, or rather oversights. Mr. Miller very properly says that "He is a sadly thoughtless and reprehensible practitioner who, in the treatment of inflammation, throws in mercury with a loose hand and a careless eye." (p. 128); but when the student reads a few lines lower down, that when mercury is indicated, "the best form of exhibition is two grains of calomel and half a grain of opium, repeated every hour or every two hours, according to the haste with which we desire to affect the system," we fear he might very likely fall into the error of supposing that this liberal administration of mercury was the rule, instead of being the rare exception, for its use in the treatment of inflammation; that cases do every now and then occur in which such heroic practice is required we do not pretend to deny, but the student should be informed that in the great majority of cases the sixth or eighth part of the quantity of mercury, above mentioned, would suffice. Such, for instance, is the case in one of the very examples adduced by Mr. Miller in the same page, as justifying "much eagerness" in giving mercury; we allude to iritis. Mr. Miller is of course perfectly right in laying it down as a general rule, that venesection should precede the administration of mercury in the treatment of inflammation, but we think he is not quite correct in saying, "should the latter" (mercury,) "go single handed in the contest, it is sure to fail; harm is done instead of good," (p. 129;) for the cases are not unfrequent in which, either from the debility of the patient, the type of disease prevailing, or the advanced period of the disease at which the practitioner is first consulted, venesection is out of the question, and mercury may and must be administered at once without any antecedent depletion. As another example of an oversight we may just mention that Mr. Miller, describing the signs indicating the formation of matter, (p. 184,) omits a local sign, namely, œdema, which is often of the greatest importance in determining the existence of suppuration, especially when deep seated. We can, however, assure our readers, that what we conceive to be errors and omissions are few indeed; and we have adduced those just mentioned, not because of their importance, or from any desire to find fault and pick holes, but as an evidence, that if we could detect a serious error we would not fail to discharge our duty by doing so.

We must find room for Professor Miller's account of the proper treatment of local gangrene, commonly called gangrena senilis; were it only for the temporary interest respecting it, excited by a late medical trial.* All scientific and experienced surgeons will (speaking generally) agree in opinion with Mr. Miller in the propriety of the discriminative treatment, so concisely yet so clearly laid down by him. It is evident from the records of the trial alluded to, that Mr. Liston's views and practice are in strict accordance with the principles laid down by our author; while, in his evidence before the court, he displayed that degree of caution and that hesitation in condemning a mode of treatment *apparently* different, which becomes the liberal and scientific surgeon. In an art so uncertain as ours, in which the proper adaptation of any particular treatment to an individual case, must be almost invariably governed by the peculiar circumstances of that case, it can very rarely happen that any practitioner is justified in stating in a court of justice that a particular treatment was positively good or positively bad, in a case not seen by himself:

"In the chronic gangrene of old people, the *Gangrena senilis*, we may have two distinct forms: death, direct, from mere want of power; or death indirect, weakened power being overcome by inflammatory action. In the former case, cautious general support is expedient, enough to maintain and increase general power; yet cautious to avoid the induction of an inflammatory process, which we know the part to be unable to bear; and the part itself may be covered over with tepid water dressing, or any other bland protective application.

"In the second, or inflammatory form—much the more frequent—our object should be to subdue the local or perverted action, yet without impairing, on the contrary rather adding to, the general power of system. The best local application with this view, is the nitrate of silver, pencilled over the red, painful, and swoln parts, so as merely to blacken, and obtain the simply sedative and antiphlogistic result; covering the part afterwards with a light soft poultice, or water-dressing. The patient should be kept in the recumbent posture, with the part somewhat elevated. The diet must be non-stimulant, otherwise action, already beyond the power of the part to bear, will be further increased; it must yet be not truly antiphlogistic, or starving, otherwise both general and local power, already weak, will be still further impaired, and the existing action, even without increase, rendered more and more destructive. It will consist then, of simple farinaceous food; such as will maintain power, and yet not favour undue vascular action. At the same time the continued use of opiates is highly expedient. Great pain and general irritation attend the progress of the disorder. The former is in part alleviated by the nitrate of silver; both will be much assuaged by opium, which further, according to some, would seem to exert a beneficial tonic effect on the capillaries, thereby tending to increase vital power under circumstances when it is much required. Under such treatment, we expect, and often not in vain, pain, redness, and swelling to cease, as also the advance of the mortification; a healthy line of demarcation is established; the dead parts are thrown off; the patient rallies greatly in his system; and in short recovery is obtained, though not of course without more or less mutilation.

"But such was not the practice, and such were not the results of former times. The practitioner took but a one-sided view of the case; regarding deficient power alone, and overlooking redundant action. His patient was literally crammed with diet of the most rich and stimulant kind; if in the better ranks of life, his table was made to groan daily, as well as himself, under the most sumptuous viands; and yet the generous food seemed only to feed the disease, not the patient. The dusky redness spread more and more, and both part and frame sank under it.

* Baker v. Lowe. Lancet, Feb. 1845.

The error was at length perceived, and an opposite extreme was gone into. Seeing then nothing but overaction, antiphlogistics were had recourse to, as if the process were of the ordinary sthenic form; disregarding the want of power, which did not fail to increase under the neglect. Dupuytren, for example, trusted to venesection. Now, a middle place is wisely selected; we neither stimulate nor spoliate the system; local action is moderated, while both local and general power is enhanced and maintained; and the result is altogether satisfactory." (pp. 269-70.)

We had marked several other passages in Mr. Miller's work for the purpose of extracting them, but must limit ourselves to the following one on the treatment of the shock that so frequently follows the receipt of an injury, for the mischief that results from ignorance of or inattention to the rules so well laid down by Mr. Miller, is we believe much greater than is perhaps commonly supposed. After distinguishing between mental and corporeal shock, Mr. Miller thus describes the treatment of the latter:

"Two errors—in their nature very much opposed—require to be guarded against; foolish bleeding, and premature stimulation. A patient having received a fall, is probably found unconscious and incapable of motion; the practitioner would seem, in some instances, to have a peculiar aptitude for mistaking such a state for the coma induced by extravasation; a vision of apoplexy with its suitable remedy of venesection, passes on the instant through his mind; his lancet, as it were mechanically, leaves its case, and reaches a vein in the bend of the arm, or the jugular vein, or the temporal artery. No blood may follow the incision; and it is well, for loss of blood—a most powerful agent of depression —is not likely to prove beneficial when depression is already great and dangerous. By and bye, reaction begins to be established; the pulse may be felt and counted, the skin becomes warm, and signs of returning consciousness appear; at this stage, bleeding is not unfrequently practised; still it is premature. Nature now, however, is in a better state of self-defence; but little of the precious fluid escapes, ere syncope again occurs, arresting the flow—a protest and a safeguard against the malapraxis. The time for bleeding is neither before reaction nor during its early progress, but after it has been fully established, and when it threatens to advance to an inflammatory excess . . . . . . In the treatment of the shock of injury then—and more especially when the head is the part injured, early bleeding and immature stimulation are both to be avoided. The injured part receives the mechanical adjustment that is necessary, the patient is laid in bed, or elsewhere, as comfortably as possible; with the head (unless it be the seat of injury) in the first instance rather low, so as to favour return of arterial circulation there. The event is then carefully watched. Reaction may soon occur without further aid from us, and require even active means for its moderation. When it is tardy and there seems to exist no reason why its retardation should be desirable, friction and heat are to be applied to the general surface; and should these fail, stimuli are then cautiously administered by the mouth; beginning with simple fluids, such as hot tea or soup, and gradually ascending in the scale if need be, to brandy and ammonia. The exhibition of these requires care when insensibility is complete; otherwise they may get into the air-passages instead of into the gullet, and suffocate the patient. Also in urgent cases, powerful stimuli—as sinapisms, hot irons, blisters, strong ammonia—may be applied to the surface, with the double object of rousing the spinal system by reflex action, and courting sanguineous circulation towards the part irritated. In the use of such remedies it is to be remembered that sensation is for the time in abeyance; and unless we, as it were, feel for the patient, the applications are apt to be unnecessarily severe; proving very troublesome, and perhaps even dangerous, in their results, after reaction has been established—as by ulceration, sloughing, or extension of superficial inflammation. The ammonia, for example, of a smelling bottle has often been carelessly thrown into the nostrils, producing sad disturbance there . . . . lives have actually been lost by nasal inflammation, so induced.

"The internal use of stimuli must also be conducted with extreme caution, and desisted from so soon as circulation is restored satisfactorily; otherwise danger by excessive reaction can scarcely be escaped. If inflammatory fever set in, along with local inflammation in the injured part, not only are all stimuli scrupulously withheld, but antiphlogistics are administered as circumstances may demand. If, on the other hand, irritative fever be the result, opium or other narcotics, in guarded doses are to be exhibited. And it is to be borne in mind that when the shock has been severe and protracted, and more especially when it has occurred in a frame previously weak, the sthenic period of reaction is apt to be but short, and the tendency is to gangrene locally, with typhoid seizure of the system; in such cases the more powerful antiphlogistics must be employed sparingly if at all. When the injury has been attended with great loss of blood, reaction is never of the sthenic form, but of the purely nervous kind, formerly described when treating of venesection; for the assuaging of this a full opiate is most effectual. Vomiting usually disappears before the ordinary restoratives, along with the other symptoms of the shock. Should it prove troublesome, as it sometimes does, with hiccup—it may be directly treated by a sinapism to the epigastric region, and small doses of the spiritus ammoniæ aromaticus." (pp. 172-5.)

From the extracts given, the reader will perceive that Mr. Miller's style, although very lively, clear, and energetic, is somewhat peculiar. It reminds us of the late John Bell's. It is a style which only a scholar and a man of talent could write; and yet we have our doubts whether it is not better suited for the lecture-room, than for a grave didactic treatise: wherefore, when Mr. Miller next appears before the profession, as an author, which we trust will be soon, we would recommend him to consider whether he would not do himself and his subject more justice, by restraining his *verve* with a stricter rein, and keeping his eloquence for his class-room. There, we doubt not, it is quite appropriate and must tell. But this is a mere matter of taste, which Mr. Miller may probably think himself as good a judge of as ourselves. It is more to the purpose to say that we cordially approve of his work, and trust it may have the success it so well deserves. It contains a sound body of surgical doctrine, and speaks well for Mr. Miller's scientific attainments. It ought to be read by all young surgeons, and not a few old.

---

